# Dietary Patterns and Age-Related Macular Degeneration in Korea: The Korea National Health and Nutrition Examination Survey 2010–2011

**DOI:** 10.1038/s41598-019-44632-2

**Published:** 2019-06-03

**Authors:** Kyung Tae Kang, Yu Cheol Kim

**Affiliations:** Department of Ophthalmology, Keimyung University School of Medicine, Dongsan Medical Center, Daegu, Korea

**Keywords:** Macular degeneration, Epidemiology

## Abstract

This study was performed to reveal dietary patterns and age-related macular degeneration risk association in general Korean population. A retrospective cross-sectional database analysis using the data collected from January 2010 to December 2011 at a Korea nationwide survey was conducted. The present analysis was performed from December 2016 to November 2017. Detailed grading with fundus photographs was performed by observers blinded to the patient characteristics. The current study focused on subjects forty year and older who had fundus photographs that is assessable from at least one eye (7,899 participants). Participants were excluded if they reported extreme energy intake (142 participants) or if they were likely to have changed dietary behavior (1,171 participants), or with missing data (n = 764). After exclusion, 5,843 participants data were analyzed in the current study. As the result, 6.8% of the participants exhibited early stages of age-related macular degeneration and 0.6% exhibited late stages. Furthermore, relatively more frequent fish consumption was associated reduced odds of early age-related macular degeneration when comparing the third quartile with the first quartile groups, however, relatively more frequent legume consumption was associated with reduced odds of late age-related macular degeneration when comparing the third quartile with the first quartile groups. In conclusion, the current study insists that the diet pattern rich in fish and legume might have protective effect against age-related macular degeneration in Korean population.

## Introduction

Age-related macular degeneration (AMD) is one of the major causes of visual impairment in industrialized countries and can cause blindness in more than 3 million people worldwide^1–4^.

This progressive, late-onset degenerative disease make patient lose the central vision and can significantly reduce quality of life^5^. The AMD occurrence increases with the age of a population, and therefore increases burden on health-care resources of industrialized countries^6–8^.

There is no proven effective treatment for early stage of AMD and late AMD except neovascular AMD. Therefore, considerable interest in identifying risk factors to prevent or delay AMD progression is present recently^9^. Numerous studies have identified age, family history, smoking, and hyperopia as risk factors for AMD^10–14^. Recently, diet and nutrition are revealed to be modifiable risk factors for AMD in the previous studies^15–17^, as healthier diets were suspected to lower AMD risk^18^. However, most studies evaluating dietary factors and AMD risk association were placed in the American and European regions^15–17,19–21^, and few studies have been performed in Asia^22–24^. Although health problems that is increases in aging countries, such as AMD, are becoming more important as the elderly population increases in Korea, epidemiologic studies evaluating the dietary factors and AMD risk association in general Korean population are scarce^18,25^. Two such studies have been performed in Korea, but they mainly focused on the intake of single nutrients or food types in each gender^18^. Assessing dietary factors and AMD risk association regarding how foods in Korean population are actually consumed in daily life has never been evaluated.

Thus, the current article was written to evaluate AMD prevalence and its association with dietary patterns by using data of a Korea nationwide survey from 2010 to 2011.

## Results

### Study participants

**Figure 1:**
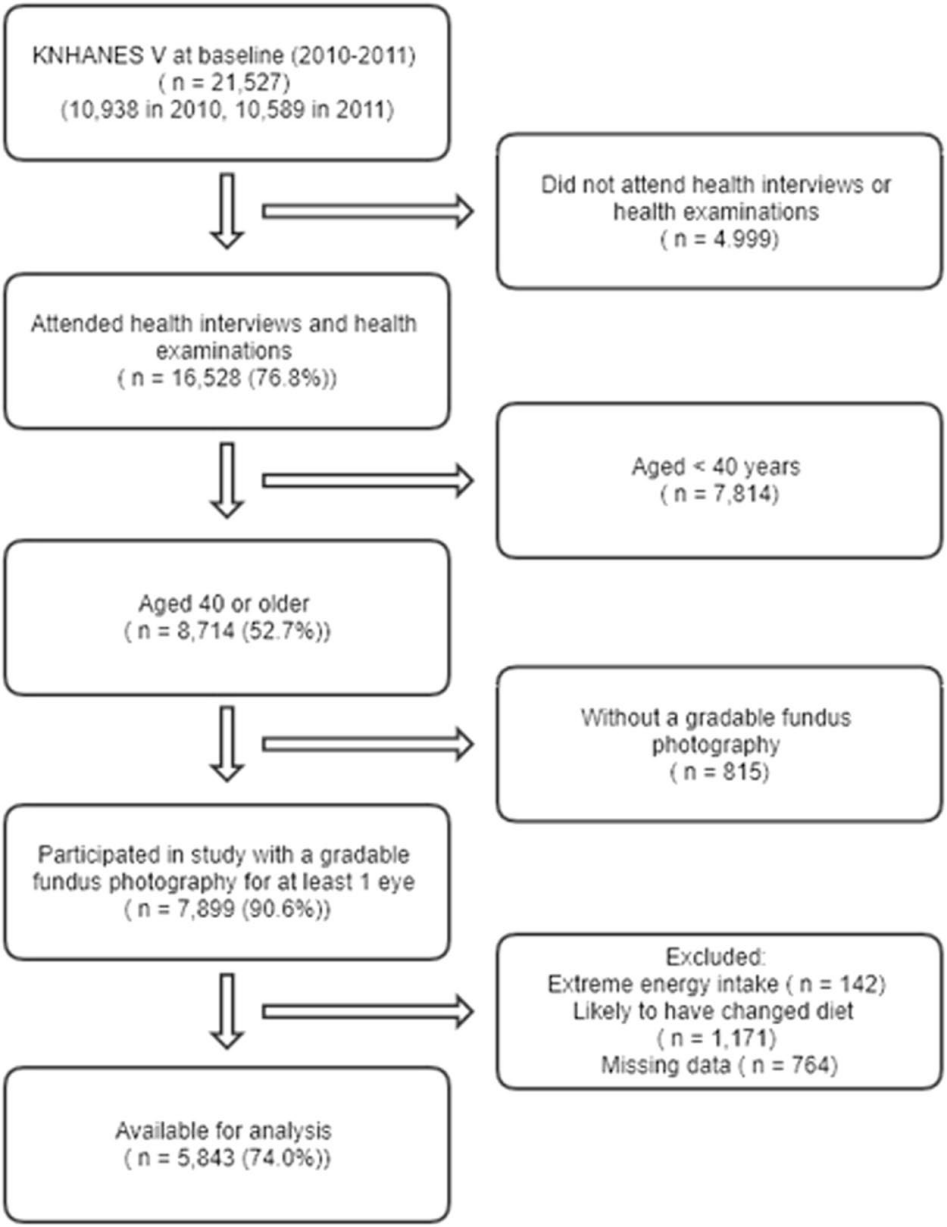
Study Participation Flowchart for KNHANES V 2010–2011. KNHANES V, the fifth Korea National Health and Nutrition Examination Survey.

Of the 21,527 eligible KNHANES V (2010–2011) participants, 16,528 attended health interviews and underwent examinations. There were 8,714 participants 40 or older, and 7,899 of them had a gradable fundus photograph. After excluding 2,056 participants with extreme energy intake (n = 142), those who were likely to have changed dietary behavior (n = 1,171), or with missing data (n = 764), 5,843 participants were analyzed (Fig. 1).

### Prevalence of overall AMD, early AMD, late AMD

Table 1 showed the weighted prevalence rates and frequencies of early AMD and late AMD. The prevalence of overall AMD was estimated at 7.5% (95% CI, 6.8–8.2). The early AMD prevalence was estimated at 6.8% (95% CI, 6.2–7.5). The late AMD prevalence was 0.6% (95% CI, 0.5–1.0), which included 0.5% (95% CI, 0.3–0.8) for wet AMD prevalence and 0.1% (95% CI, 0.1–0.4) for geographic atrophy prevalence. The early AMD prevalence was 1.4% (95% CI, 0.3–2.2) in those 40 to 49 years old, 5.0% (95% CI, 4.0–6.2%) in those 50 to 59 years old, 13.0% (95% CI, 11.2–15.1%) in those 60 to 69 years old, and 17.8% (95% CI, 15.5–20.5%) in those 70 and older. The prevalence of early AMD increased with age. The similar trend was observed in the late AMD prevalence rate.

**Table 1:** Weighted prevalence rates and frequencies of early and late age-related macular degeneration in South Korea, based on the KNHANES V (2010–2011).

	All AMD	Early AMD	Late AMD		
			All late AMD	Wet AMD	Geographic Atrophy
Mean age (95% CI)	63.7 (62.4–65.1)	63.6 (62.2–65.1)	64.7 (60.5–68.8)	63.8 (59.1–68.5)	68.8 (60.4–77.3)
Overall % (95% CI)	7.5 (6.8–8.2)	6.8 (6.2–7.5)	0.6 (0.5–1.0)	0.5 (0.3–0.8)	0.1 (0.1–0.4)
Frequency	660	607	54	41	13
**Age groups (years)**					
40–49, % (95% CI)	1.5 (1.0–2.3)	1.4 (0.3–2.2)	0.1 (0.0–0.6)	0.1 (0.0–0.6)	0
Frequency	26	24	2	2	0
50–59, % (95% CI)	5.6 (4.5–6.9)	5.0 (4.0–6.2)	0.6 (0.3–1.4)	0.5 (0.2–1.2)	0.1 (0.0–0.8)
Frequency	120	110	10	9	1
60–69, % (95% CI)	14.2 (12.3–16.4)	13.0 (11.2–15.1)	1.2 (0.6–2.3)	0.9 (0.4–1.8)	0.3 (0.1–1.2)
Frequency	232	216	16	12	4
≥70, % (95% CI)	19.5 (17.1–22.2)	17.8 (15.5–20.5)	1.7 (1.1–2.5)	1.1 (0.7–1.8)	0.7 (0.3–1.4)
Frequency	282	257	26	18	8

*AMD*, age-related macular degeneration; *CI*, confidence interval; *KNHANES V*, the fifth Korea National Health and Nutrition Examination Survey.

**Figure 2:**
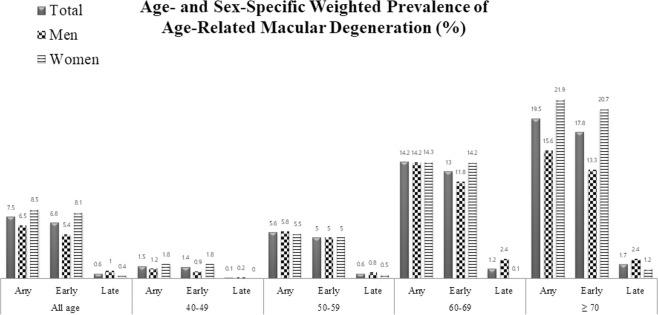
Age- and Sex-specific Weighted Prevalence of Any, Early, and Late Age-Related Macular Degeneration in South Korea based on the KNHANES V (2010–2011). KNHANES V, the fifth Korea National Health and Nutrition Examination Survey.

Figure 2 exhibit the age- and sex-specific weighted early and late AMD prevalence. The early AMD prevalence was lower in men than in women across the all age groups, with the exception of the 50 to 59 group, in which both sexes demonstrated a similar prevalence. The late AMD prevalence was higher in men than in women in all age groups.

### Early age-related macular degeneration and multiple logistic regression analyses

Associations between mean intake frequencies of dietary groups and early AMD are shown in Table 2 with multivariable-adjusted odd ratios. Early AMD showed similar results in the model 1 and 2. The meat group was associated with a statistically significant difference among each quartile group (p < 0.05 in models 1 and 2), but the results reveal no definite odds change when compared with the first quartile. The fish group was associated with a 43% decrease in odds for early AMD in model 1 and a 39% decrease in model 2 after adjustment for covariates in the third quartile (OR, 0.57; 95% CI, 0.38–0.84 in model 1, OR, 0.61; 95% CI, 0.40–0.92 in model 2). However, the odds for the fish group were not reduced for the second and fourth quartiles. Although ORs showed downward trend across quartiles for early AMD in model 2 in the fruit group, analysis of the other food groups did not show any statistically significant association.

**Table 2:** Odds ratios for early age-related macular degeneration according to the mean intake frequency of dietary groups in South Korea, based on the KNHANES V (2010–2011).

Dietary groups, number of the patients (%)	Range of mean intake frequency per week	Multivariable model 1^a^			Multivariable model 2^b^		
		*Odds ratio*	95% *CI*	P *value*^c^	*Odds ratio*	95% *CI*	P *value*^c^
**Group 1: cereals**							
First quartile, 163 (26.85)	<3.72	1.00	Reference	0.37	1.00	Reference	0.32
Second quartile, 139 (22.90)	3.72–4.94	0.89	0.62–1.28		0.81	0.56–1.18	
Third quartile, 155 (25.54)	4.94–6.15	0.77	0.52–1.16		0.73	0.47–1.11	
Fourth quartile, 150 (24.71)	>6.15	1.03	0.71–1.49		0.95	0.64–1.41	
**Group 2: legumes**							
First quartile, 162 (26.69)	<0.69	1.00	Reference	0.14	1.00	Reference	0.09
Second quartile, 138 (22.73)	0.69–1.75	1.28	0.87–1.88		1.39	0.92–2.09	
Third quartile, 134 (22.08)	1.75–3.65	1.62	1.08–2.44		1.75	1.14–2.70	
Fourth quartile, 173 (28.50)	>3.65	1.37	0.91–2.06		1.44	0.92–2.26	
**Group 3: meat**							
First quartile, 263 (43.33)	<1.80	1.00	Reference	0.05	1.00	Reference	0.03
Second quartile, 192 (31.63)	1.80–2.60	1.28	0.94–1.74		1.36	0.99–1.88	
Third quartile, 104 (17.13)	2.60–3.40	0.82	0.52–1.29		0.85	0.53–1.65	
Fourth quartile, 48 (7.91)	>3.40	0.74	0.42–1.28		0.78	0.45–1.37	
**Group 4: fish**							
First quartile, 223 (36.74)	<0.31	1.00	Reference	0.01	1.00	Reference	0.02
Second quartile, 154 (25.37)	0.31–0.61	0.96	0.70–1.33		0.97	0.69–1.36	
Third quartile, 114 (18.78)	0.61–1.01	0.57	0.38–0.84		0.61	0.40–0.92	
Fourth quartile, 116 (19.11)	>1.01	1.03	0.66–1.60		1.23	0.72–1.77	
**Group 5: vegetables**							
First quartile, 194 (31.96)	<1.88	1.00	Reference	0.37	1.00	Reference	0.46
Second quartile, 164 (27.02)	1.88–2.54	1.04	0.73–1.46		1.03	0.71–1.50	
Third quartile, 123 (20.26)	2.54–3.24	0.76	0.53–1.11		0.77	0.52–1.14	
Fourth quartile, 126 (20.76)	>3.24	0.96	0.66–1.41		0.92	0.62–1.39	
**Group 6: seaweeds**							
First quartile, 206 (33.94)	<0.62	1.00	Reference	0.68	1.00	Reference	0.64
Second quartile, 127 (20.92)	0.62–1.79	1.00	0.72–1.41		0.99	0.70–1.42	
Third quartile, 162 (26.69)	1.79–3.00	1.15	0.82–1.63		1.10	0.77–1.56	
Fourth quartile, 112 (18.45)	>3.00	0.91	0.62–1.35		0.84	0.55–1.26	
**Group 7: fruit**							
First quartile, 236 (38.88)	<0.27	1.00	Reference	0.14	1.00	Reference	0.15
Second quartile, 139 (22.90)	0.27–0.53	0.72	0.50–1.02		0.71	0.49–1.04	
Third quartile, 134 (22.08)	0.53–0.90	0.74	0.51–1.06		0.68	0.46–1.00	
Fourth quartile, 98 (16.14)	>0.90	0.62	0.40–0.96		0.66	0.42–1.02	
**Group 8: dairy products**							
First quartile, 242 (39.87)	<0.16	1.00	Reference	0.92	1.00	Reference	0.93
Second quartile, 124 (20.43)	0.16–0.60	0.92	0.63–1.64		0.96	0.66–1.41	
Third quartile, 129 (21.25)	0.60–2.08	1.03	0.73–1.46		1.05	0.74–1.49	
Fourth quartile, 112 (18.45)	>2.08	1.04	0.73–1.49		1.09	0.75–1.57	
**Group 9: drinks**							
First quartile, 204 (33.61)	<1.91	1.00	Reference	0.40	1.00	Reference	0.67
Second quartile, 171 (28.17)	1.91–3.33	0.85	0.63–1.15		0.91	0.66–1.26	
Third quartile, 146 (24.05)	3.33–5.86	0.98	0.70–1.38		1.09	0.76–1.57	
Fourth quartile, 86 (14.17)	>5,86	0.74	0.50–1.11		0.85	0.55–1.31	
**Group 10: alcohol**							
First quartile, 369 (60.8)	0.00	1.00	Reference	0.81	1.00	Reference	0.96
Second quartile, 22 (3.60)	0.00–0.08	1.02	0.53–1.93		0.95	0.48–1.85	
Third quartile, 91 (15.00)	0.08–0.72	0.88	0.64–1.22		0.91	0.65–1.28	
Fourth quartile, 125 (20.60)	>0.72	1.08	0.76–1.55		0.98	0.67–1.43	
**Group 11: snacks**							
First quartile, 405 (66.72)	0.00	1.00	Reference	0.93	1.00	Reference	0.93
Second quartile, 90 (14.83)	0.00–0.08	0.93	0.64–1.35		0.95	0.64–1.39	
Third quartile, 16 (2.64)	0.08–0.19	1.20	0.51–2.80		1.24	0.54–2.85	
Fourth quartile, 96 (15.81)	>0.19	1.04	0.69–1.55		1.05	0.70–1.59	

*CI*, confidence interval; KNHANES V, the fifth Korea National Health and Nutrition Examination Survey.

^a^Age, sex, smoking status (never, former, or current), education (with or without graduation from middle school), body mass index (more or less than 25 kg/m^2^), waist circumference (more or less than 90 cm in men and 80 cm in women), total energy intake adjusted.

^b^Age, sex, smoking status (never, former, or current), education (with or without graduation from middle school), body mass index (more or less than 25 kg/m^2^), waist circumference (more or less than 90 cm in men and 80 cm in women), total energy intake, anemia (more or less than 13 g/dL in men or 12 g/dL in women), gamma-glutamyl transferase, high-density lipoprotein, hepatitis B surface antigen adjusted.

^c^Multiple logistic regression analysis.

### Late age-related macular degeneration and multiple logistic regression analyses

Table 3 shows associations between mean intake frequencies of dietary groups and late AMD with multivariable-adjusted odd ratios. The legume group was associated with statistically significant differences among quartile groups (p < 0.05 in model 2), and a 69% decrease in odds for late AMD in model 2 in the third quartile (OR, 0.21; 95% CI, 0.05–0.93). Although ORs showed upward trend across quartiles for late AMD in the meat group, analysis of other food groups did not show any statistically significant association.

**Table 3:** Odds ratios for late age-related macular degeneration according to the mean intake frequency of dietary groups in South Korea, based on the KNHANES V (2010–2011).

Dietary groups, number of the patients (%)	Range of mean intake frequency per week	Multivariable model 1^a^			Multivariable model 2^b^		
		*Odds ratio*	95% *CI*	P *value*^c^	*Odds ratio*	95% *CI*	P *value*^c^
**Group 1: cereals**							
First quartile, 14 (25.93)	<3.72	1.00	Reference	0.99	1.00	Reference	0.99
Second quartile, 9 (16.67)	3.72–4.94	0.92	0.27–3.15		0.95	0.26–3.41	
Third quartile, 16 (29.63)	4.94–6.15	0.96	0.36–2.58		0.93	0.31–2.78	
Fourth quartile, 15 (27.77)	>6.15	0.95	0.29–3.12		0.91	0.25–3.31	
**Group 2: legumes**							
First quartile, 16 (29.63)	<0.69	1.00	Reference	0.05	1.00	Reference	0.02
Second quartile, 15 (27.78)	0.69–1.75	1.21	0.42–3.44		1.33	0.53–3.30	
Third quartile, 5 (9.26)	1.75–3.65	0.23	0.05–1.11		0.21	0.05–0.93	
Fourth quartile, 18 (33.33)	>3.65	0.49	0.17–1.43		0.53	0.18–1.53	
**Group 3: meat**							
First quartile, 18 (33.33)	<1.80	1.00	Reference	0.83	1.00	Reference	0.74
Second quartile, 15 (27.78)	1.80–2.60	1.31	0.48–3.54		1.38	0.51–3.79	
Third quartile, 13 (24.07)	2.60–3.40	1.60	0.58–4.43		1.67	0.58–4.80	
Fourth quartile, 8 (14.82)	>3.40	1.63	0.44–6.09		1.99	0.52–7.61	
**Group 4: fish**							
First quartile, 18 (33.33)	<0.31	1.00	Reference	0.08	1.00	Reference	0.06
Second quartile, 17 (31.48)	0.31–0.61	1.70	0.67–4.34		1.56	0.60–4.08	
Third quartile, 8 (14.82)	0.61–1.01	0.44	0.13–1.46		0.38	0.09–1.47	
Fourth quartile, 11 (20.37)	>1.01	0.99	0.33–3.01		0.81	0.26–2.52	
**Group 5: vegetables**							
First quartile, 14 (25.93)	<1.88	1.00	Reference	0.69	1.00	Reference	0.60
Second quartile, 13 (24.07)	1.88–2.54	1.79	0.64–5.04		1.94	0.71–5.33	
Third quartile, 12 (22.22)	2.54–3.24	1.48	0.46–4.80		1.68	0.48–5.89	
Fourth quartile, 15 (27.78)	>3.24	1.92	0.55–6.68		2.09	0.57–7.68	
**Group 6: seaweeds**							
First quartile, 12 (22.22)	<0.62	1.00	Reference	0.43	1.00	Reference	0.26
Second quartile, 19 (35.19)	0.62–1.79	1.41	0.48–4.12		1.45	0.51–4.14	
Third quartile, 11 (20.37)	1.79–3.00	0.83	0.25–2.80		0.87	0.25–2.96	
Fourth quartile, 12 (22.22)	>3.00	1.93	0.67–5.52		2.18	0.74–6.39	
**Group 7: fruit**							
First quartile, 18 (33.33))	<0.27	1.00	Reference	0.88	1.00	Reference	0.89
Second quartile, 12 (22.22)	0.27–0.53	0.87	0.33–2.31		0.83	0.30–2.26	
Third quartile, 13 (24.07)	0.53–0.90	1.20	0.43–3.37		1.14	0.42–3.07	
Fourth quartile, 11 (20.38)	>0.90	1.54	0.45–5.20		1.46	0.40–5.34	
**Group 8: dairy products**							
First quartile, 20 (37.04)	<0.16	1.00	Reference	0.73	1.00	Reference	0.52
Second quartile, 11 (20.37)	0.16–0.60	1.67	0.54–5.12		1.75	0.59–5.18	
Third quartile, 11 (20.37)	0.60–2.08	1.03	0.37–2.91		0.92	0.33–2.59	
Fourth quartile, 12 (22.22)	>2.08	1.52	0.56–4.14		1.82	0.64–5.15	
**Group 9: drinks**							
First quartile, 13 (24.08)	<1.91	1.00	Reference	0.10	1.00	Reference	0.11
Second quartile, 15 (27.78)	1.91–3.33	1.25	0.44–3.53		1.55	0.48–4.93	
Third quartile, 18 (33.33)	3.33–5.86	0.88	0.24–3.20		1.05	0.27–3.99	
Fourth quartile, 8 (14.81)	>5,86	0.23	0.04–1.67		0.29	0.05–1.78	
**Group 10: alcohol**							
First quartile, 28 (51.85)	0.00	1.00	Reference	0.56	1.00	Reference	0.45
Second quartile, 3 (5.56)	0.00–0.08	1.21	0.24–6.16		1.04	0.20–5.30	
Third quartile, 5, (9.26)	0.08–0.72	0.38	0.10–1.52		0.33	0.08–1.36	
Fourth quartile, 18 (33.33)	>0.72	0.81	0.28–2.36		0.57	0.18–1.79	
**Group 11: snacks**							
First quartile, 30 (55.56)	0.00	1.00	Reference	0.45	1.00	Reference	0.35
Second quartile, 11 (20.37)	0.00–0.08	1.57	0.53–4.66		1.65	0.59–4.63	
Third quartile, 2 (3.70)	0.08–0.19	3.39	0.71–21.22		4.27	0.79–23.03	
Fourth quartile, 11 (20.37)	>0.19	1.72	0.62–4.77		1.94	0.68–5.53	

*CI*, confidence interval; KNHANES V, the fifth Korea National Health and Nutrition Examination Survey.

^a^Age, sex, smoking status (never, former, or current), education (with or without graduation from middle school), body mass index (more or less than 25 kg/m^2^), waist circumference (more or less than 90 cm in men and 80 cm in women), total energy intake adjusted.

^b^Age, sex, smoking status (never, former, or current), education (with or without graduation from middle school), body mass index (more or less than 25 kg/m^2^), waist circumference (more or less than 90 cm in men and 80 cm in women), total energy intake, anemia (more or less than 13 g/dL in men or 12 g/dL in women), gamma-glutamyl transferase, high-density lipoprotein, hepatitis B surface antigen adjusted.

^c^Multiple logistic regression analysis.

## Discussion

This study reported AMD prevalence and its association with dietary patterns by using data from KNHANES V from 2010 to 2011. Based on the result, the prevalence rates of early AMD was 6.8%, and it of late AMD was 0.6% in South Korea. These rates are nearly identical to the previous results in a meta-analysis of Asian populations, which estimated the prevalence rates of early AMD at 6.8% and that of late AMD at 0.4%^26^. The prevalence rates of AMD in the present study are also similar to rates reported in a Survey (6.5% and 0.8%, respectively) conducted in the United States^27^. In a recent meta-analysis revealed the prevalence of AMD in Asian populations were quite similar to the it of AMD in western countries^28^. These studies result shows that there is no prominent difference of the prevalence of AMD among inter-ethnic groups^26–28^. The early and late AMD prevalence rates of Korean population were estimated as 6.7% and 0.7% in a previous study that investigated the results of KNHANES V^29^; the estimate from the prior study was not consistent with the current study, although both studies investigated the same data, because the current study used dietary factor-associated exclusion criteria. Nonetheless, the estimated AMD prevalence rates were nearly identical because of the composite sample analysis methods, and these rates are assumed to represent the general Korean population.

Some previous studies show that late AMD prevalence in men is higher than women^25,29^, while other studies have shown the opposite results^30–32^. Further, some studies have reported there is no difference in the prevalence of early or late AMD after controlling for age depends on sex^13,33,34^. Therefore, the difference of AMD prevalence in men and women have not fully revealed yet. This difference may come from the higher prevalence of polypoidal choroidal vasculopathy or high smoker prevalence in Asian men^28,33,35^. The AMD prevalence also increased steadily as the age of the participants increased. This may suggest an association between cumulative aging-related changes and the development of AMD.

Many previous studies have shown that a healthy diet, such as the Mediterranean diet, provides relatively high amounts of bioactive antioxidant compounds^36^. It is well-known that healthy diets are essential to limit the cognitive and physical degeneration during aging process^37^. Association of dietary patterns with systemic diseases, such as cardiovascular disease or cancer, also have been reported^38–40^. Nevertheless, assessing the dietary factors and AMD risk association analysis has not been fully discussed yet. There have been previous studies, mainly performed in Western countries, assessing the association between dietary patterns and AMD. A Study showed that lower prevalence of early AMD was associated with Mediterranean diet^41^. An analysis of the Healthy Eating Index and Alternative Healthy Eating Index (AHEI) showed that advanced AMD is related to overall diet quality^42^. The Melbourne Collaborative Cohort Study demonstrated that a diet low in red meat and high in fruits, vegetables, chicken, and nuts is also associated AMD prevalence^43^. Similarly, an Oriental diet pattern was known to decrease odds of AMD, whereas a Western diet pattern increased AMD odds ratio^44^. A study was performed by using the FFQ and the alternate Mediterranean diet score with data. The results supported the conclusion that Mediterranean diet pattern may reduce the progression risk of advanced AMD. Another study showed that people with Mediterranean diet pattern have higher concentration of beneficial biomarkers than those without^36^.

The findings of the previous studies are similar to the results of our dietary patterns analysis. In the current study, we observed that in the fish group, the third quartile demonstrated a 43% and 39% decrease in odds for early AMD in model 1 and 2, respectively, compared with the first quartile. Long-chain omega-3 fatty acids which is rich in fish, have been known to have protective effects against cardiovascular disease in some studies^45^. A relationship between intake of omega-3 fatty acids and the progression of AMD is biologically plausible because there is a high level of omega-3 fatty acids in retina^46^. Consumption of fish were associated with lower risk of developing AMD in observational studies^47–49^. We also observed that in the legume group, the third quartile had a 69% decrease in odds for late AMD in model 2, compared with the first quartile. Legumes contain significant amounts of macular carotenoids, including lutein and zeaxanthin^50^. Carotenoids give retina protection against toxins or damage, and absorb and filter blue light, which is harmful to RPE cells^51–53^. A study revealed a relationship between carotenoids and advanced AMD, but not between carotenoids and intermediate AMD; this was consistent with the results of the present study^54^. Another study insisted that lutein- and zeaxanthin-rich diets can reduce intermediate risk AMD in women^55^. Consumption of these carotenoids has been proven to delay AMD progression to advanced stage from intermediate stage of AMD in some studies^56,57^. The effectiveness of carotenoids in preventing AMD seems to exist, but the effects can differ depends on its phase^18^.

We noticed that different dietary groups were associated with different type of AMD. This may be because diets have a different degree of influence with regard to primary prevention and secondary prevention^58^. Some dietary factors may promote or prevent drusen accumulation or disturbance of retinal pigment. On the other hand, others may induce or prevent neoangiogenesis in AMD^59^. Therefore, dietary factors may do not influence early AMD in the same manner as they do in late AMD. This difference may also be due to other variables, such as genetic variation or confounders not included in this study. Further research may help clarify associations with diet patterns. Fruit intake had a decreased odds ratio of early AMD in our result but the association was not significant. Similarly, meat intake had an increased odds ratio of late AMD but this association was not significant.

Our analysis has a strength in its design because we grouped dietary factors according to how they were actually consumed. The association between AMD risk and specific dietary factor is hard to assess because people do not consume foods and nutrients as solitary form. There are also synergistic relationships between food components^37,41^. We evaluated dietary factors and mean intake frequencies of dietary groups based on FFQs, and compared each quartile with the first quartile. We believe that this is a simple way to assess the association and to compare the results with other studies. Other strength is the current study has large size of sample, standardized data collection, and multiple-step AMD grading methods. Maintaining consistency with prior studies assessing AMD risk and dietary pattern reduces the chance that the current results are due to chance. Also, the current study was designed to minimize residual confounding by including anemia, GGT, HDL and HBsAg, which were indicated in previous risk assessment studies^25,29^.

This study has some limitations includes the fact that the nature this study, which is cross-sectional study, limits its strength. Also, we measured the diet pattern based on a single FFQ. This may not be able to represent long-term, lifelong consumption. However, we excluded subjects with previous diagnoses likely associated with dietary change, and the variations among days were not known to be different significantly^18^. Since the study only included the data of the subject with gradable fundus photo to assess the AMD, this might induce selection bias and have an effect on making inaccurate estimation of AMD prevalence. Some may also be concerned that the method of assessing dietary patterns included only frequency, and not the amount of intake. However, assessing quantitative diet along with frequency of food intake has been included in KNHANES since 2012. Data from 2012 and during 2010–2011 were not comparable because the structure of data had been modified. Additionally, the ocular examination with AMD grading has been suspended since 2013. Therefore, we believed the correct way to assess is to include two years of data from the 2010–2011 survey, as this includes larger samples from the general population. In addition, the statistical significance of results comparing first and third quartiles in fish and legume groups remains unclear. We considered whether the proportions of the fourth quartiles in fish and legume groups might be extremely small, but they were not. Although the data analysis included total energy intake as a covariate in multiple logistic regression, fourth quartile groups may have tendency to consume other dietary factors more frequently than others. It is also suspected to be partially because we did not evaluate quantities of food, but only evaluated the frequency of dietary intake in the current study. We suggest enrolling more participants by including results from more than two years of data collection, in order to determine whether the results show statistical significance in other quartiles, compared with the first quartile, in further studies.

In conclusion, our results revealed that more frequent fish intake is possibly associated with lower risk of early AMD, and more frequent legumes intake is possibly associated with lower risk of late AMD. Despite potential genetic differences and different AMD prevalence rates in Western countries and South Korea, a healthy dietary pattern is likely to have association with a lower AMD risk in both regions. This current study cannot determine the exact amounts of certain foods required, but the results further support the opinion that dietary pattern intervention is another modifiable factor to prevent development or progress of AMD.

## Methods

### Study design

The current study was based on the results of the KNHANES, which was a population, and cross-sectional based survey, administered to the general population of South Korea. The survey included ophthalmologic examinations from 2008 to 2012. In order to keep the measurement of items homogenous across the data, the present study analyzed 2010 and 2011 data from the KNHANES V. Because there were some modifications in the 2009 and 2010 surveys regarding measurement of low-density lipoprotein and gamma-glutamyl transferase (GGT). Moreover, after 2012 dietary factors were assessed using a semi-quantitative method instead of the food frequency questionnaires (FFQ) survey method used previously^25^.

In the KNHANES V, a total of 3,840 households in 192 districts were selected annually with a sampling design that used strata, cluster, and weight^18^. The response rates were 81.9% and 80.4% in 2010 and 2011, respectively. All family members >1 year of age in each selected household were included as subjects (21,527 participants). All subjects participated in the health interview, including the dietary pattern assessment, and health and ophthalmologic examinations. In the current study, participants 40 or older that had a fundus photograph available for analysis from at least one eye (7,899 participants) were included for analysis.

Informed consent was obtained, and KCDC institutional review board approved the process of the survey (IRB no. 2010-02CON-21-C, 2011-02CON-06-C)^18^. The authors have conducted this study regarding Declaration of Helsinki. The KNHANES component included the health interview, health examination, and the survey of nutrition. The details of the health examination and the health interview survey performed in the KNHANES has been covered widely in the related previous studies^18,25^.

### Dietary data assessment

The KNHANES survey collected dietary data by a form of FFQ which included 63-items, recorded as a single-day 24-hour recall. On the interview day, participants were asked to report any food and drinks consumed during a day before. Daily energy intake was calculated using the FFQ result. We excluded those who reported extreme energy intakes (less than 1^st^ percentile or more than 99^th^ percentile, 142 participants), which suggested that the FFQ had been improperly completed, or were likely to have changed their diet because of predisposed diseases (1,171 participants), or with missing data (n = 764). Finally, 5,843 participants were included for study analysis. Food types in the FFQ were reassigned as 11 different food groups. Detailed food group information was well listed in a previous study^18^. The mean intake frequencies of dietary groups were calculated as the average frequency of each food intake in a certain group. The mean intake frequencies were then analyzed by quartile grouping.

### Statistical analysis and covariates definitions

Smoking status was classified as “never smoker”, “former smoker”, or “current smoker”. Education status was classified into two groups: with or without graduation from middle school. BMI was divided into two groups: participant with a BMI of more or less than 25 kg/m^2^. According to their WC, two groups were defined: those with a WC of less than or more than 90 cm or 80 cm, respectively in men or women. The survey recorded anemia if a subject’s hemoglobin level was less than 13 g/dL or 12 g/dL, in men or women respectively; this was measured with an XE-2100D analyzer (Sysmex, Kobe, Japan). Hepatitis B surface antigen (HBsAg) (electrochemiluminescence immunoassay E-170, Roche, Mannheim, Germany), lipoproteins (enzymatic cholesterol assay Automatic Analyzer 7600, Hitachi, Tokyo, Japan), and GGT (Enzymatic, Pureauto SGGT, Sekisui, Japan) were also measured^18,29^.

All statistical analyses were performed with the Statistical Package for the Social Sciences version 19.0.1 (SPSS Inc, Chicago, IL, USA) by using strata, cluster, and weight variables for composite sample analysis. The KNHANES sample weight was adjusted for oversampling and nonresponse^18^. Statistical significance was defined when *P* values was less than 0.05.

The mean intake frequencies of dietary groups were analyzed based on approximate quartile grouping, with the first quartile used as the reference group. Odds ratios (ORs) for each type of AMD, according to mean intake frequencies of dietary groups, were established by multivariable logistic regression with 95% confidence intervals (CIs). Model 1 analyzed well-established risk factors of AMD: age, sex, smoking, education, BMI, WC, and total energy intake. To minimize potential confounders, the risk factors suggested in recent studies prior to KNHANES (i.e., anemia, GGT, high-density lipoprotein (HDL), and HBsAg), were included in the Model 2 analysis^25,29^.
